# Tumour-necrosis factor from the rabbit. III. Relationship to interferons

**DOI:** 10.1038/bjc.1979.218

**Published:** 1979-10

**Authors:** N. Matthews

## Abstract

Tumour-necrosis factor (TNF) is growth-inhibitory or cytotoxic to certain tumour cell lines, and is present in the serum of rabbits injected i.v. with BCG and endotoxin 2 weeks apart (TNF serum). TNF serum also has interferon activity, and as TNF and interferons have a number of properties in common their relationship has been investigated further. TNF was assayed by cytotoxicity *in vitro* against L cells and interferon by a CPE-inhibition assay with Semliki Forest virus.

TNF appears not to be an interferon, on the following bases:

1. TNF activity could be separated from the Type I interferon of TNF serum by passage through a Cibacron blue-agarose column or by sequential salt precipitation, ion-exchange chromatography and gel filtration.

2. Preparations of Type I interferon induced by poly I, poly C or virus lacked TNF activity.

3. Though it was not possible to compare TNF with rabbit Type II interferon (as methods used to induce Type II interferon in other species were unsuccessful in the rabbit) rabbit TNF has a number of properties which distinguish it from the Type II interferons of other species.

4. Rabbit TNF inhibited the growth of a human melanoma cell line, and also had effects on certain mouse and rabbit cell lines, whereas the anti-cellular effects of interferons are reported to be species-specific.


Br. J. Cancer (1979) **40**, 534

### TUMOUR-NECROSIS FACTOR FROM THE RABBIT III. RELATIONSHIP TO INTERFERONS

N. MATTHEWS

*From the Department of Medical Microbiology, Welsh National School of Medicine, Cardiff*

Received 12 February 1979 Accepted 25 June 1979

**Summary.—Tumour-necrosis factor (TNF) is growth-inhibitory or cytotoxic to certain tumour cell lines, and is present in the serum of rabbits injected i.v. with BCG and endotoxin 2 weeks apart (TNF serum). TNF serum also has interferon activity, and as TNF and interferons have a number of properties in common their relationship has been investigated further. TNF was assayed by cytotoxicity *in vitro* against L cells and interferon by a CPE-inhibition assay with Semliki Forest virus.**

**TNF appears not to be an interferon, on the following bases:**

**1. TNF activity could be separated from the Type I interferon of TNF serum by passage through a Cibacron blue–agarose column or by sequential salt precipitation, ion-exchange chromatography and gel filtration.**

**2. Preparations of Type I interferon induced by poly I, poly C or virus lacked TNF activity.**

**3. Though it was not possible to compare TNF with rabbit Type II interferon (as methods used to induce Type II interferon in other species were unsuccessful in the rabbit) rabbit TNF has a number of properties which distinguish it from the Type II interferons of other species.**

**4. Rabbit TNF inhibited the growth of a human melanoma cell line, and also had effects on certain mouse and rabbit cell lines, whereas the anti-cellular effects of interferons are reported to be species-specific.**

TUMOUR-NECROSIS FACTOR (TNF) is a substance which causes necrosis of some transplantable tumours (Carswell *et al.*, 1975) and is cytotoxic or growth-inhibitory *in vitro* to a number of cell lines (Carswell *et al.*, 1975; Matthews & Watkins, 1978). TNF is released into the blood after endotoxin injection in animals which have been pretreated with agents such as Bacillus Calmette–Guérin (BCG) or *Corynebacterium parvum*, which induce macrophage hyperplasia. There is both indirect (Old, 1976) and direct evidence (Matthews, 1978) that TNF is a product of mononuclear phagocytes.

In addition to their anti-viral effects, interferons can be growth-inhibitory or cytotoxic *in vitro* to certain tumour cell lines (Gresser, 1977; Kuwata *et al.*, 1976). Rabbit TNF and interferon have certain other properties in common, *viz.* (a) similar physicochemical characteristics, (b) production *in vivo* after challenge with endotoxin, and (c) release from mononuclear phagocytes *in vitro* after short incubation periods at 37°C but not at 4°C nor on cell disruption (Smith & Wagner, 1967; Matthews, 1978).

In this paper, the relationship between rabbit TNF and interferons is examined further.

### MATERIALS AND METHODS

*TNF production.*—TNF was obtained from rabbits given 2 i.v. injections 2 weeks apart of BCG ($50–250 \times 10^6$ organisms) and endotoxin (100 $\mu$g). The animals were bled immediately before the endotoxin injection (control

Correspondence to Dr N. Matthews, Department of Medical Microbiology, Welsh National School of Medicine, Cardiff CF4 4XN, U.K.

serum) and then 2 h later (TNF serum) unless stated otherwise. BCG was Glaxo percutaneous and endotoxin was lipopolysaccharide B or W from *E. coli* 055-B5 (Difco).

*Cell lines.*—The NK 1–4 line of human melanoma cells was derived by Dr R. H. Whitehead (Dept. of Surgery, Welsh National School of Medicine). The other cell lines have been described previously (Matthews & Watkins, 1978).

*TNF assay.*—In Sterilin M29ARTL microplates, 75 μl of target-cell suspensions ($10^5$/ml) was mixed with 75 μl of TNF or control serum dilutions, 8 replicates for each dilution. The culture medium was Eagle's minimum essential medium with 20% foetal calf serum. After incubation (usually for 4 days) at 37°C in 95% air, 5% $CO_2$, the target cells were washed ×2 with phosphate-buffered saline (PBS) pH 7·2, fixed with methanol and stained with Giemsa. An assessment of the number of cells in each well was made by locating the centre of the well at low magnification, changing to the high-power objective and counting all the cells in a constant area of the field at ×800 magnification. The % growth inhibition was calculated from the formula $100(a-b)/a$ where $a$ and $b$ are the mean number of cells in wells with, respectively, control and TNF serum. In some experiments cytotoxicity was measured photometrically, as described for the interferon assay. The two methods correlated closely except above 90% cytotoxicity, where the photometric assay was non-linear.

The titre of TNF in a given serum is defined as the reciprocal of the dilution which causes a 50% reduction in L cell numbers after 4 days of culture.

*Interferon production.*—Rabbits were injected i.v. with 0·1 mg poly I, poly C and bled after 2 h.

*Interferon assay.*—A quantitative cytopathic effect (CPE) inhibition method was used. RK 13 cells (75 μl) at $5\times10^5$/ml were seeded into the wells of Sterilin microplates and, after attachment to the plastic, 75 μl interferon dilutions were added, 6–8 replicates for each dilution. After overnight incubation at 37°C in 95% air, 5% $CO_2$, the culture medium was decanted and the cells were washed ×2 with PBS. A 1/3000 dilution(150 μl) of a stock preparation of Semliki Forest virus (SFV) was then added to each well before incubation for a further 3 days. The SFV was kindly provided by Dr N. B. Finter (Wellcome Research Laboratories, Beckenham, Kent) and, at the dilution used, over 90% of the cells were killed over the assay period in the absence of interferon. After fixation with 5% formaldehyde, the remaining cells were stained with 50% Giemsa and washed well in running water. An estimate of the cell density in each well was obtained photometrically with a Leitz inverted microscope fitted with an Orthomat automatic camera. Each well was photographed in turn using the trial-exposure setting and the exposure time was measured with a stopwatch. The settings were: film speed, 3 DIN and tungsten-lamp voltage, 4V. At the magnification used (×2·5 objective) ~60% of the area of the well was within the photographic field. Mean exposure times were calculated for each interferon dilution and the % protection against viral CPE was calculated from the equation $100(b-c)/(a-c)$ where $a$, $b$, and $c$ are the mean exposure times of wells with, respectively, virus-free medium, interferon + virus, medium + virus. Under the conditions used, the exposure time for wells with virus was about 6 s, compared to 11 s without virus, and the standard deviations were within the range 5–10% for cells without virus and 10–25% for cells with virus.

The interferon titre was calculated as the reciprocal of the dilution which inhibited the virus CPE by 50%. One interferon unit corresponds to 0·2 units of standard rabbit interferon (GO19-902-528).

A subline of RK13 cells relatively resistant to the cytotoxic effect of TNF was selected for the interferon assay.

*Affinity chromatography on Cibacron blue–agarose.*—Agarose (Pharmacia Sepharose 6B) was conjugated with Cibacron blue as described by Angal & Dean (1977). Serum samples (50 μl) were applied to a 5ml column equilibrated with PBS and run at a 20 ml/h. Unlike human or mouse albumin, rabbit albumin does not bind to Cibacron blue and therefore does not inhibit interferon binding.

*Phytohaemagglutinin (PHA) stimulation.*—Rabbit blood mononuclear leucocytes obtained by Hypaque–Ficoll sedimentation (Matthews, 1978) were suspended at $5\times10^6$/ml in MEM with 20% autologous serum with or without PHA (Wellcome reagents) at a final concentration of 1/160. After 8 h at 37°C the supernatants were removed and stored at −20°C, and the cells were replenished with fresh medium with or without

PHA as appropriate. After a further 64h incubation the supernatants were removed and stored at −20°C until required.

### RESULTS

Sera from rabbits injected sequentially with BCG and endotoxin (TNF serum) had TNF titres as measured by cytotoxicity against L cells of 5000–25,000 and interferon titres in the range 3000–5000. However, serum from a rabbit injected with the interferon inducer poly I, poly C had no cytotoxic or growth-inhibitory effects on any of the TNF-sensitive cell lines tested, despite having an interferon titre of 1160. Similarly, the standard preparation of rabbit interferon induced by Blue Tongue virus had no growth-inhibitory effects on 2 TNF-sensitive rabbit lines (RK 13 and SIRC) or on mouse L cells. From analogy with mouse interferons the above preparations would be expected to be predominately of Type I (Youngner, 1977).

As expected of a Type I interferon, the interferon activity of TNF serum proved relatively resistant to acid, retaining 50% of its activity after exposure to pH 2 for 24 h. The TNF activity was more severely affected, being reduced to 10% of the control value.

Mouse interferons can bind to Cibacron blue (de Maeyer-Guignard *et al.*, 1977) and we have found that this is also true of rabbit interferon. Passage of rabbit TNF serum through a column of Cibacron blue–agarose reduced the interferon titre about 16-fold, but not the TNF titre (Fig. 1).

Further evidence that the TNF and interferon in TNF serum are separate entities is that TNF purified sequentially by ammonium sulphate precipitation, ion-exchange chromatography and gel filtration (Matthews & Watkins, 1978) had an interferon titre of < 160 but a TNF titre of 5120.

Human and murine Type II interferons have been amply described in the literature, but there are few if any references to rabbit Type II interferon. Type II interferons are produced by lymphocytes stimulated specifically with antigen or non-specifically with mitogens, and they are more acid-labile and have less antiviral activity than Type I interferons (see Youngner, 1977). Mice primed with BCG and injected 2–3 weeks later with endotoxin produce Type I interferon in maximal amounts 2 h after injection; if challenged instead with tuberculin antigen, Type II interferon is produced maximally 6 h later (Youngner & Salvin, 1978).

FIG. 1.—(a) Interferon and (b) TNF assay for (▲) TNF serum and (△) TNF serum passed through a Cibacron-blue–agarose column.

In an attempt to compare TNF with rabbit Type II interferon, a rabbit injected $2\frac{1}{2}$ weeks previously with BCG ($50–250 \times 10^6$ organisms) was further injected i.v. with PPD (50,000 u) and bled immediately before and 2 h and 6 h after the PPD injection. Only low amounts of interferon were produced, with titres of 10 and 5 at 2 h and 6 h respectively. TNF-like activity was present in the 2h sample at a low titre (1/50) but not in the pre-

TABLE I.—*Comparison of TNF and interferon production by rabbit mononuclear leucocytes cultured with or without PHA*

| | TNF titre Time (h) | | Interferon titre Time (h) | |
|---|---|---|---|---|
| | 0–8 | 8–72 | 0–8 | 8–72 |
| Medium only | 80 | 12 | 2 | un* |
| Medium + PHA | 40 | 12 | 9 | un |

* un = undetectable.

injection or 6h bleeds. Although this experiment has not clarified the relationship between Type II interferon and TNF, it indicates that TNF is not produced by a BCG-primed rabbit on challenge with specific antigen (PPD).

Type II interferons can also be produced *in vitro* by human or mouse mononuclear leucocytes stimulated by PHA, maximal amounts being produced after 1–3 days in culture (Epstein, 1977). Table I shows that rabbit mononuclear leucocytes cultured with PHA produced undetectable amounts of interferon over the 8–72h period of culture, and only small amounts of TNF; the cultures were glycolytically active and showed microscopic evidence of lymphocyte transformation. Over the first 8 h of culture more significant amounts of TNF and interferon were produced, irrespective of whether PHA was present. TNF is a product of the monocytes in the culture (Matthews, 1978) and this cell type may well be the source of the interferon (Smith & Wagner, 1967). This experimental approach has also failed to illuminate the relationship between Type II interferon and TNF.

As reported previously, rabbit TNF is cytotoxic to certain mouse cell lines, as well as to a rabbit cell line. Rabbit TNF is also growth-inhibitory to a second rabbit cell line (SIRC) and to a human melanoma cell line (Fig. 2). No effect has been found on 2 other melanoma cell lines or on a further 6 human lines of other histological types.

Release from unstimulated rabbit mononuclear phagocytes of TNF (Matthews, 1978) and interferon (Smith & Wagner,

FIG. 2.—Effect of (▲) purified TNF (diluted 1/20 with medium) or (△) medium alone on the growth of human NK1–4 melanoma cells.

1967) occurs after similar incubation periods *in vitro*. The following experiment was performed to see whether these entities are released in parallel *in vivo*. A rabbit injected with BCG 2 weeks previously was bled immediately before (0 h) and ½, 1 and 2 h after endotoxin injection, and the sera were tested for interferon and TNF activity. Because of the shocked state of the rabbit, the experiment was terminated at 2 h. From Table II it can be seen that

TABLE II.—*Serum concentrations of TNF and interferon in a BCG-primed rabbit at various times after endotoxin injection*

| | Titre | |
|---|---|---|
| Time (h) | TNF | Interferon |
| 0 | < 160 | < 160 |
| ½ | 2028 | < 160 |
| 1 | 9433 | 2144 |
| 2 | 12500 | 3348 |

the concentration of both substances is greatest 2 h after endotoxin challenge. TNF but not interferon is detectable ½ h after challenge, although this may simply reflect the greater sensitivity of the TNF assay.

After i.v. injection, interferon is rapidly eliminated from the blood (Ho, 1973). This is also true of TNF as after injection of 1 ml TNF serum i.v. into a normal

FIG. 3.—TNF activity of serum from a normal rabbit taken (■) just before, (▲) 2 min after or (△) 60 min after i.v. injection of 1 ml TNF serum.

rabbit, no TNF was detectable in the serum 1 h later (Fig. 3).

### DISCUSSION

Interferons are heterogeneous and have been much better characterized in man and the mouse than in the rabbit. Human and murine interferons can be classified as Type I, induced by viruses and various synthetic inducers, and Type II, released from lymphocytes activated by specific antigens or mitogens. TNF is clearly distinct from Type I, since preparations expected to contain predominantly Type I interferon (induced by poly I, poly C or Blue Tongue virus) lacked TNF activity. On the basis of its acid stability, and from analogy with the mouse (Youngner & Salvin, 1978) the BCG/endotoxin-induced interferon in TNF serum is probably Type I. TNF was distinguishable from the interferon component of TNF serum by its greater lability to acid, and the fact that the activities were largely separable by passage through a Cibacron-blue column or by sequential salt precipitation, ion-exchange chromatography and gel filtration. The association between TNF and Type II interferon is less clear. We have been unable to find any reference to rabbit Type II interferon in the literature, and our attempts to induce it either *in vivo* or *in vitro* have been unsuccessful. Arguing solely from analogy with human and murine Type II interferons, TNF differs from Type II interferon. Thus, unlike Type II interferon, TNF production is provoked by injection of endotoxin but not PPD into a BCG-primed animal. Also, rabbit mononuclear leucocytes in tissue culture produce TNF within the first 8 h of culture with or without the addition of PHA, whereas Type II interferon requires the presence of PHA, and is produced later in culture. In tissue culture, TNF is produced by monocytes (Matthews, 1978) and Type II interferon predominantly by lymphocytes (Epstein, 1977).

As well as the resemblance in physico-chemical characteristics and comparable conditions of synthesis *in vitro* by mononuclear phagocytes remarked upon previously, TNF and interferon have a similar rate of release after endotoxin injection into a BCG-primed rabbit, and both substances are rapidly removed from the circulation after i.v. injection.

Only some cell lines are affected by TNF, and there is no correlation with the production of C-type or other viruses, tumorigenicity, growth rate or mycoplasma contamination (Matthews & Watkins, 1978, and unpublished observations). Of 25 continuous cell lines tested so far from a number of species, only 9 are susceptible to TNF. Primary cultures of human, mouse or hamster fibroblasts or rabbit kidney cells are not susceptible.

TNF is clearly a separate entity from Type I interferon and probably distinct from Type II interferon, yet they have a number of common properties. Furthermore, TNF and interferons can modulate immune responses (Gresser, 1977; Hoffman *et al.*, 1978) and inhibit granulocyte/macrophage colony formation *in vitro* (Shah *et al.*, 1978) suggesting perhaps a regulatory role in immunity and inflammation.

I thank Mrs M. L. Neale for capable technical assistance and Dr H. C. Ryley for provision of the Cibacron-blue–agarose.

### REFERENCES

ANGAL, S. & DEAN, P. D. G. (1977) The effect of matrix on the binding of albumin to immobilised Cibacron Blue. *Biochem. J.*, **167,** 301.

CARSWELL, E. A., OLD, L. J., KASSELL, R. L., GREEN, S., FIORE, N. & WILLIAMSON, B. (1975) An endotoxin-induced serum factor that causes necrosis of tumor. *Proc. Natl Acad. Sci U.S.A.*, **72,** 3666.

DE MAEYER-GUIGNARD, J., THANG, M. N. & DE MAEYER, E. (1977) Binding of mouse interferon to polynucleotides. *Proc. Natl Acad. Sci. U.S.A.*, **74,** 3787.

EPSTEIN, L. B. (1977) Mitogen and antigen induction of interferon *in vitro* and *in vivo*. *Tex. Rep. Biol. Med.*, **35,** 42.

GRESSER, I. (1977). On the varied biologic effects of interferon. *Cell Immunol.*, **24,** 406.

HO, M. (1973) Pharmaco-kinetics of interferons. In *Interferon and Interferon Inducers*, Ed. Finter. Amsterdam: North-Holland Pub. Co. p. 241.

HOFFMAN, M. K., OETTGEN, H. F., OLD, L. J., MITTLER, R. S. & HAMMERLING, U. (1978) Induction and immunological properties of tumor necrosis factor. *J. Reticuloendothel. Soc.*, **23,** 307.

KUWATA, T., FUSE, A. & MORINAGA, N. (1976) Effects of interferon on cell and virus growth in transformed human cell lines. *J. Gen. Virol.*, **33,** 7.

MATTHEWS, N. (1978) Tumour-necrosis factor from the rabbit. II. Production by monocytes. *Br. J. Cancer*, **38,** 310.

MATTHEWS, N. & WATKINS, J. F. (1978) Tumour-necrosis factor from the rabbit. I. Mode of action, specificity and physicochemical properties. *Br. J. Cancer*, **38,** 302.

OLD, L. J. (1976) Tumor necrosis factor. *Clin. Bull.*, **6,** 118.

SHAH, R. G., GREEN, S. & MOORE, M. A. S. (1978) Colony stimulating and inhibiting activities in mouse serum after *Corynebacterium parvum* endotoxin treatment. *J. Reticuloendothel. Soc.*, **23,** 29.

SMITH, T. J. & WAGNER, R. F. (1967) Rabbit macrophage interferons. I. Conditions for biosynthesis by virus-infected and uninfected cells. *J. Exp. Med.*, **125,** 559.

YOUNGNER, J. S. (1977) Properties of interferon induced by specific antigens. *Tex. Rep. Biol. Med.*, **35,** 17.

YOUNGNER, J. S. & SALVIN, S. B. (1978) Type I and II interferons and migration inhibition factor: production in *Mycobacterium bovis* BCG-infected mice desensitised with old tuberculin or lipopolysaccharide. *Infect. Immun.*, **19,** 912.


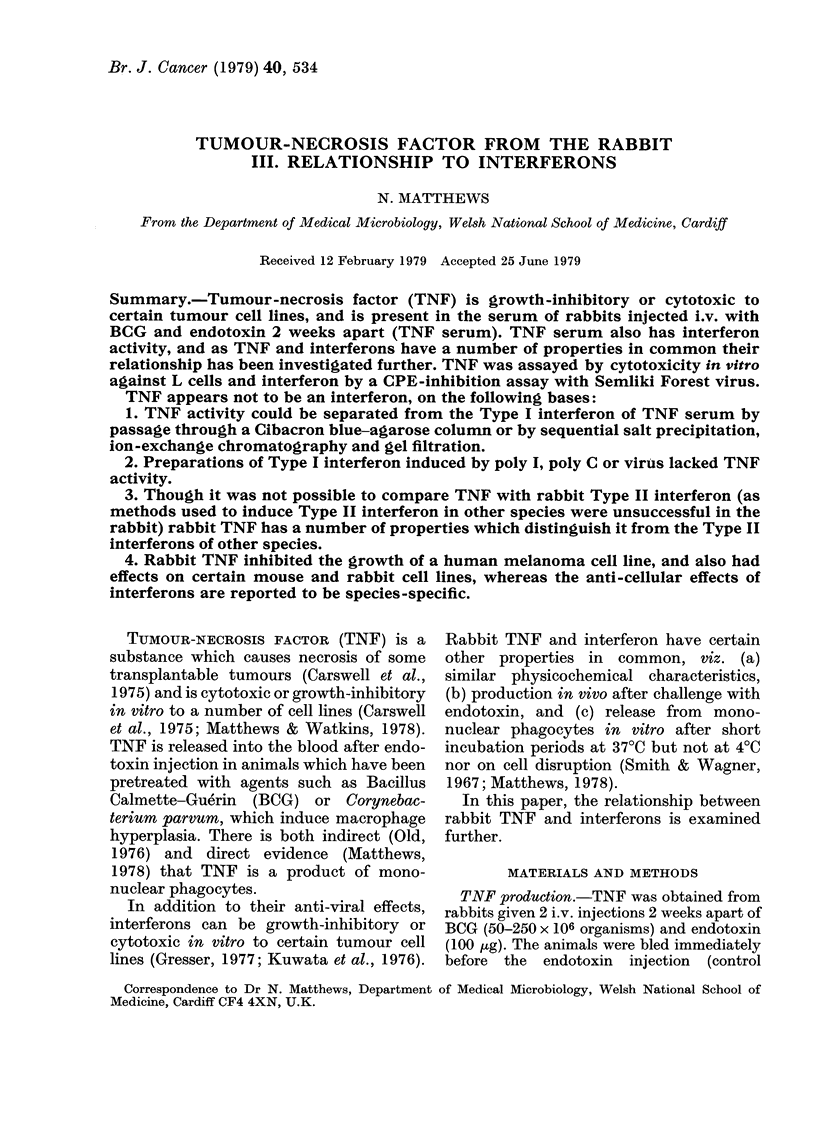

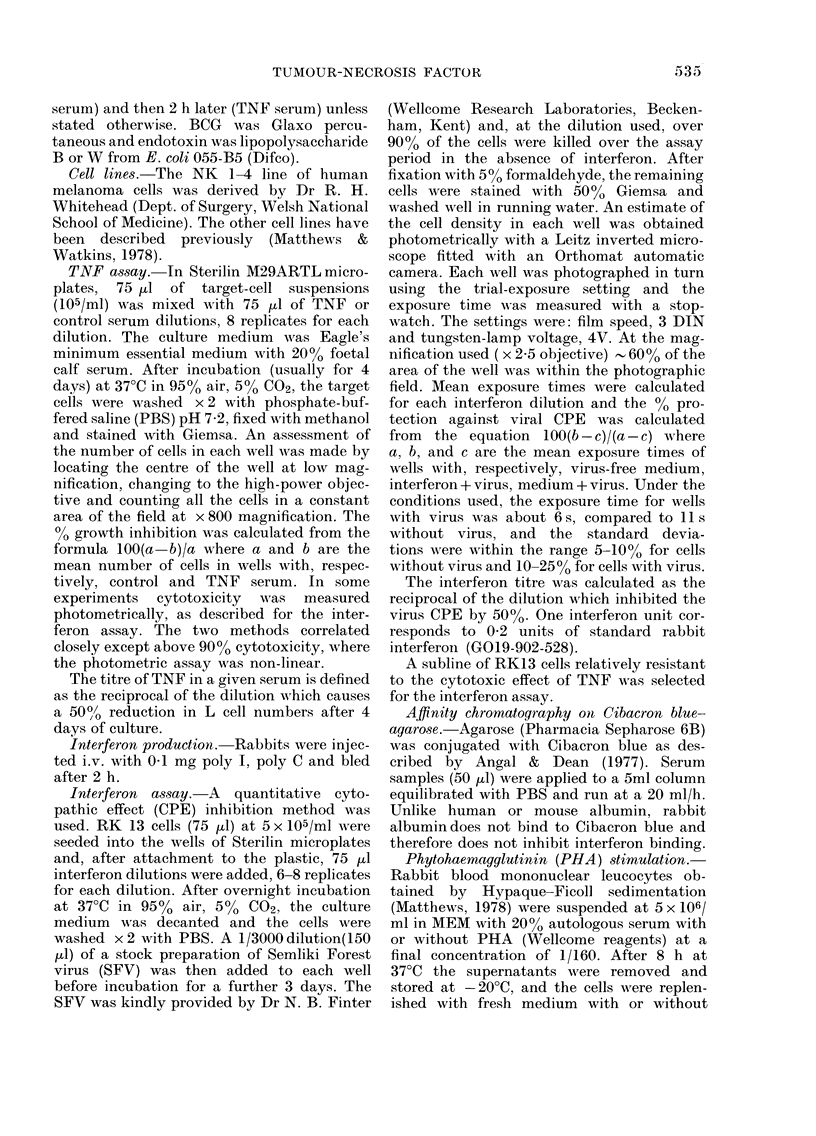

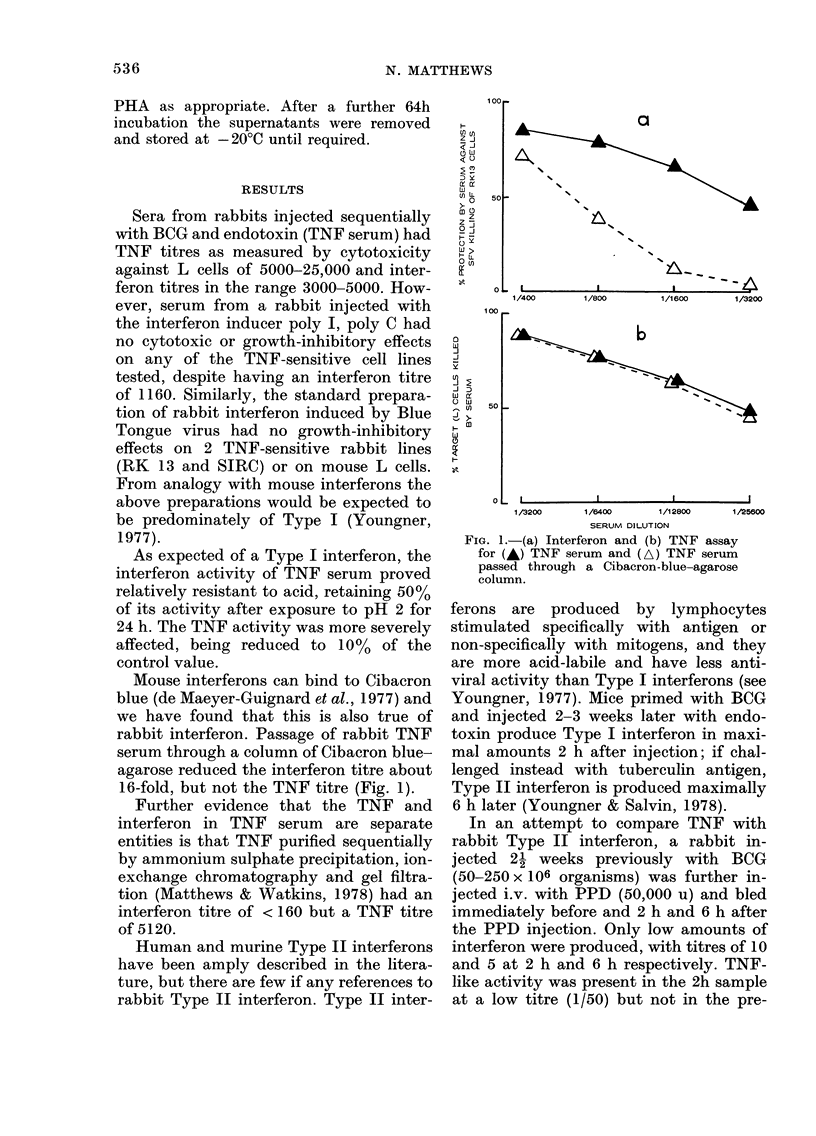

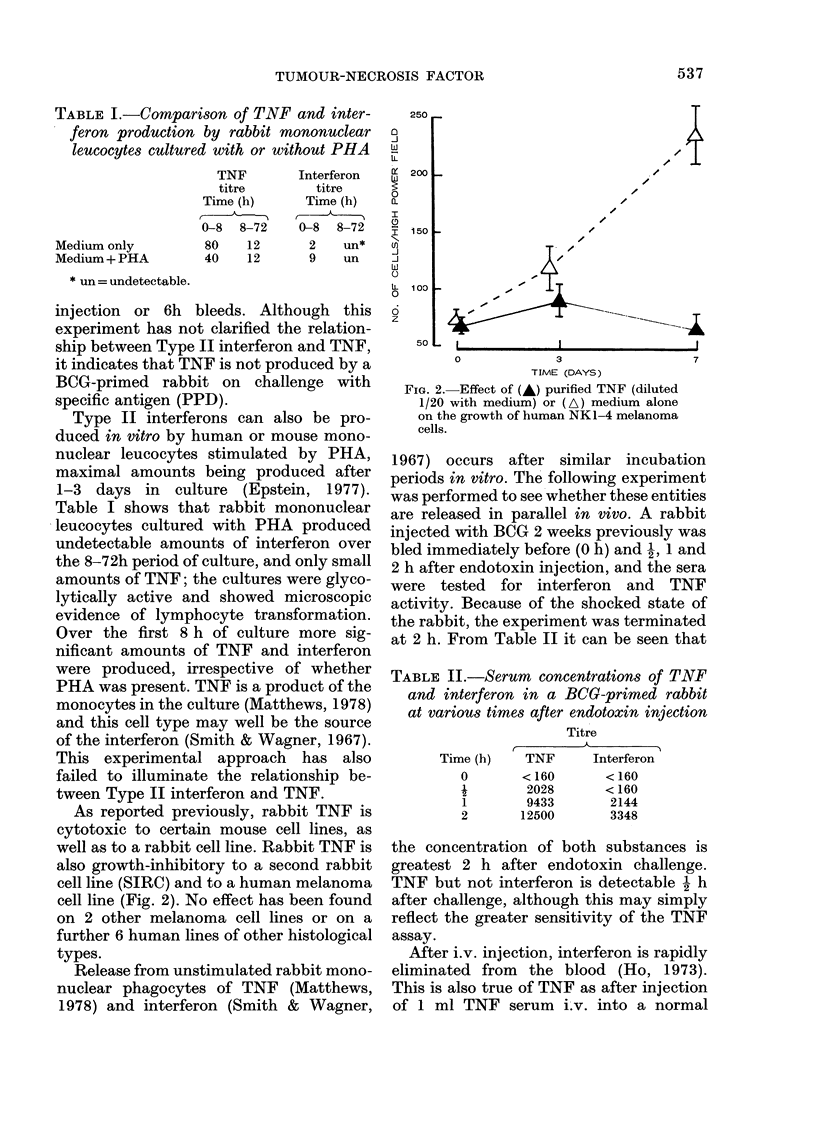

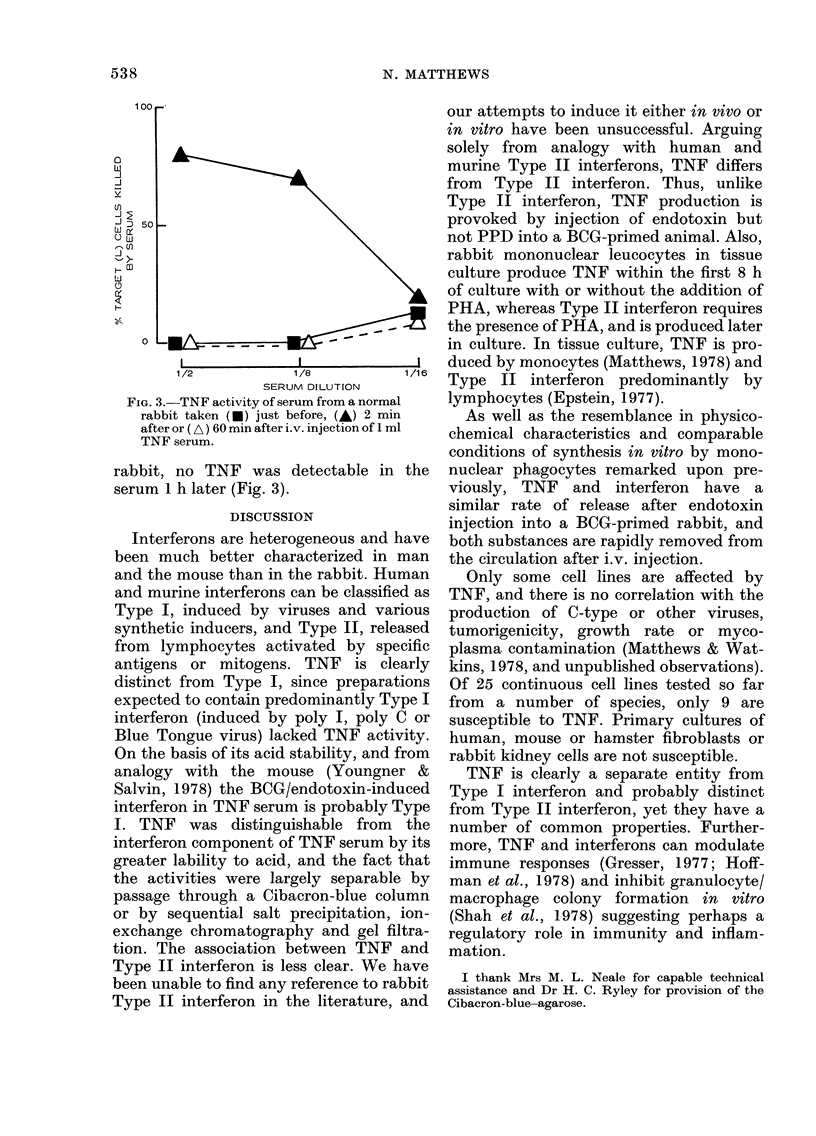

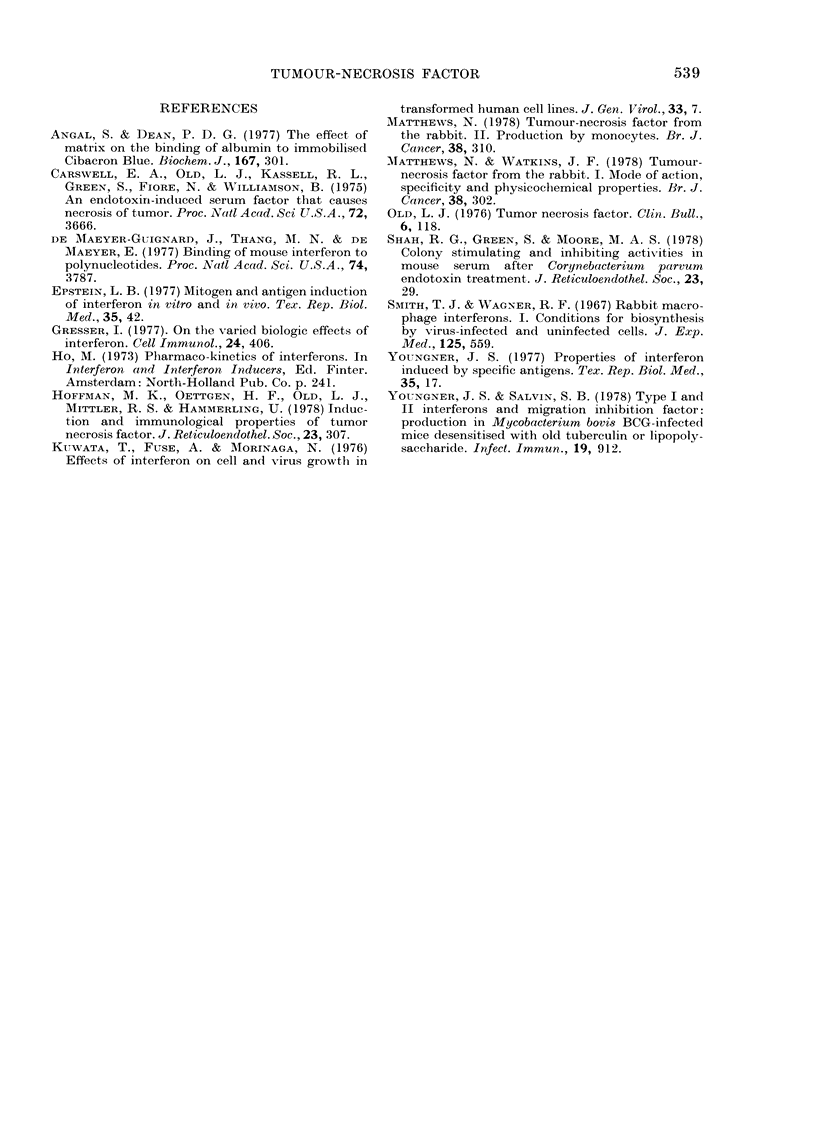


## References

[OCR_00688] Angal S., Dean P. D. (1977). The effect of matrix on the binding of albumin to immobilized Cibacron Blue.. Biochem J.

[OCR_00693] Carswell E. A., Old L. J., Kassel R. L., Green S., Fiore N., Williamson B. (1975). An endotoxin-induced serum factor that causes necrosis of tumors.. Proc Natl Acad Sci U S A.

[OCR_00702] De Maeyer-Guignard J., Thang M. N., De Maeyer E. (1977). Binding of mouse interferon to polynucleotides.. Proc Natl Acad Sci U S A.

[OCR_00706] Epstein L. B. (1977). Mitogen and antigen induction of interferon in vitro and in vivo.. Tex Rep Biol Med.

[OCR_00726] Fuse T. K., Morinaga N. (1976). Effects of interferon on cell and virus growth in transformed human cell lines.. J Gen Virol.

[OCR_00711] Gresser I. (1977). On the varied biologic effects of interferon.. Cell Immunol.

[OCR_00720] Hoffmann M. K., Oettgen H. F., Old L. J., Mittler R. S., Hammerling U. (1978). Induction and immunological properties of tumor necrosis factor.. J Reticuloendothel Soc.

[OCR_00731] Matthews N. (1978). Tumour-necrosis factor from the rabbit. II. Production by monocytes.. Br J Cancer.

[OCR_00736] Matthews N., Watkins J. F. (1978). Tumour-necrosis factor from the rabbit. I. Mode of action, specificity and physicochemical properties.. Br J Cancer.

[OCR_00742] Old L. J. (1976). Tumor necrosis factor.. Clin Bull.

[OCR_00746] Shah R. G., Green S., Moore M. A. (1978). Colony stimulating and inhibiting activities in mouse serum after Corynebacterium parvum-endotoxin treatment.. J Reticuloendothel Soc.

[OCR_00753] Smith T. J., Wagner R. R. (1967). Rabbit macrophage interferons. I. Conditions for biosynthesis by virus-infected and uninfected cells.. J Exp Med.

[OCR_00759] Youngner J. S. (1977). Properties of interferon induced by specific antigens.. Tex Rep Biol Med.

[OCR_00764] Youngner J. S., Salvin S. B. (1978). Type I and II interferons and migration inhibitory factor: production in Mycobacterium bovis BCG-infected mice desensitized with old tuberculin or lipopolysaccharide.. Infect Immun.

